# Meta-analysis of gene signatures and key pathways indicates suppression of JNK pathway as a regulator of chemo-resistance in AML

**DOI:** 10.1038/s41598-021-91864-2

**Published:** 2021-06-14

**Authors:** Parastoo Modarres, Farzaneh Mohamadi Farsani, Amir Abas Nekouie, Sadeq Vallian

**Affiliations:** 1 Department of Cell and Molecular Biology and Microbiology, Faculty of Science and Technology, University of Isfahan, Isfahan, Islamic Republic of Iran; 2 Saied Al-Shohada Hospital, Isfahan University of Medical Sciences, Isfahan, Islamic Republic of Iran; 3 Present Address: Department of Biology, Naghshejahan Higher Education Institute, Isfahan, Islamic Republic of Iran

**Keywords:** Cancer, Computational biology and bioinformatics, Genetics, Molecular biology

## Abstract

The pathways and robust deregulated gene signatures involved in AML chemo-resistance are not fully understood. Multiple subgroups of AMLs which are under treatment of various regimens seem to have similar regulatory gene(s) or pathway(s) related to their chemo-resistance phenotype. In this study using gene set enrichment approach, deregulated genes and pathways associated with relapse after chemotherapy were investigated in AML samples. Five AML libraries compiled from GEO and ArrayExpress repositories were used to identify significantly differentially expressed genes between chemo-resistance and chemo-sensitive groups. Functional and pathway enrichment analysis of differentially expressed genes was performed to assess molecular mechanisms related to AML chemotherapeutic resistance. A total of 34 genes selected to be differentially expressed in the chemo-resistance compared to the chemo-sensitive group. Among the genes selected, *c-Jun, AKT3, ARAP3, GABBR1, PELI2* and *SORT1* are involved in neurotrophin, estrogen, cAMP and Toll-like receptor signaling pathways. All these pathways are located upstream and regulate JNK signaling pathway which functions as a key regulator of cellular apoptosis. Our expression data are in favor of suppression of JNK pathway, which could induce pro-apoptotic gene expression as well as down regulation of survival factors, introducing this pathway as a key regulator of drug-resistance development in AML.

## Introduction

Acute myeloid leukemia (AML) is one of the most aggressive, life-threatening hematological malignancies characterized by uncontrolled proliferation of abnormal differentiated and nonfunctional myeloid precursor cells^1^. Clonal expansion of ≥ 20% malignant myeloblasts in the peripheral blood, bone marrow and other tissues has been considered as an indication for diagnosis of AML^2^. The incidence of AML as the most common type of acute leukemia increases substantially with age and leads to impaired hematopoietic system^3^. According to their clinical and genetic features including age, performance status, molecular/cytogenetic alterations and organ functions, a variety of cytotoxic chemotherapy agents are used in treatment regimens for AML patients^4^.

The common standard induction therapy for AML has not yet changed for more than four decades, consisting of sequential courses of a combination of anthracycline for 3 days and cytarabine (cytosine arabinoside, Ara-C), for 7–10 days (“7 + 3” regimen)^5^. Some of the clinicians intensify induction therapy by adding an anthracycline or other therapeutic compounds to enhance the likelihood of achieving a complete remission (CR)^6^. These alterations in the standard frontline therapy include use of different types of anthracyclines, mainly daunorubicin, idarubicin and etoposide as well as different cytotoxic agents such as topoisomerase II inhibitors (mitoxantrone), nucleoside analogues (azacitidine) and gemtuzumab ozogamicin (GO; a CD33-directed antibody-drug conjugate) which are added to induction therapy with or without cytokines and differentiation agents^4,6,7^.

All of these cytotoxic compounds have DNA-damaging effects that make tumor cells more susceptible to death. These anti-leukemic regimens represent strong activity against cell cycle progression and proliferation in multiple ways, including intercalating to DNA and termination of DNA synthesis, DNA damaging and inhibition of DNA replication^7–10^.

Despite acceptable rate of initial CR of 60–80% in adult younger than 65 years and 40–60% in older adult with > 65 years old, a major therapeutic challenge in all cases is drug resistance due to recurrent refractory and relapse during therapy^11^. This indicates an indispensable need for the development of novel targeted therapies through further investigation to identify chemo-resistance-regulated gene signatures and focus on pathways that are restricted to resistance of AML cases to cytotoxic agents.

In this study we performed cross-platform meta-analyses of several public microarray-based datasets contributed to chemotherapeutic response in AML to identify robust gene-expression signatures and pathways associated with drug resistance. First significantly differentially expressed genes between chemo-resistance and chemo-sensitive groups were investigated. Then, receiver operating characteristic (ROC) analysis was used to evaluate the predictive ability of differentially expressed genes (DEGs). Functional and pathway enrichment analysis of DEGs were also performed to provide deeper understanding of molecular mechanism of DNA damaging-induced chemotherapy resistance.

## Results

### Data collection and filtering

Five publicly accessible microarray datasets consisting of 131 arrays in total were matched to our predetermined inclusion criteria. The datasets were as following: (1) GSE52919 involved a gene expression profiling of patients with AML receiving chemotherapy with cytarabine (Ara-C) and daunorubicin (DNR) gene expression. The participants of this microarray dataset were adult with the age of 18–61 years with median age of 39 years, (2) GSE52891 contained expression profiling associated with pediatric relapsed AML patients with median age of 13.2 after receiving cytarabine and anthracycline as an initial therapy, (3) GSE75086 consisted of RNA expression profiling of samples with AraC-based chemotherapy at post induction, relapse and diagnostic sample (Of these, the relapsed samples were used), (4) GSE107465 encompassed expression profiling of 30 different AML patients who received different chemotherapy protocols. Among these patients, those treated with cytarabine, anthracyclines and other DNA-damaging agents such as daunorubicin, idarubicin, mitoxantrone, azacitidine and gemtuzumab ozogamicin were selected. The age of participants of this microarray dataset were ranged from 19 to 84 years with median age of 51 years, (5) GSE45249 contained gene expression profiling of three chemo-resistant subpopulation of leukemic stem cells (LSCs) to daunorubicin and cytarabine from 9 patients with primary childhood AML (27 samples in total). The detailed information of these five datasets was also provided in Table 1.

**Table 1:** Characteristics of the gene expression datasets included in the meta-analysis.

Accession no	Platform	Drugs	Tissue	Sample	Age	Number of samples		
						Selected/total	Sensitive	Resistance
GSE52919	GPL13252; Agilent	Daunorubicin and Cytarabine	Bone marrow	Patient	Adult	12/15	8	4
GSE52891	GPL570: Affymetrix	Cytarabine and Anthracycline^a^	Bone marrow/peripheral blood	Patient	Childhood	17/23	–	17
GSE75086	GPL16686 : Affymetrix	Cytarabine	Peripheral blood	Patient	NA	4/36	–	4
GSE107465	GPL570: Affymetrix	MEC, Anthracycline, Cytarabine, Azacitidine, GO	Peripheral blood	Patient	Adult	9/30	6	3
GSE45249	GPL571; Affymetrix	Daunorubicin and Cytarabine	Bone marrow	Patient’s cells	Childhood	11/27	–	11

*MEC* Mitoxantrone, Etoposide, Cytarabine, *Go* Gemtuzumab ozogamicin, *NA* not available.

^a^Anthracycline: Daunorubicin, Idarubicin, Etoposide (VP16), Mitoxantrone.

After removing the outliers and irrelevant sample arrays, the normalized datasets composed of 53 sample arrays were obtained for further downstream analysis. Then, the samples were classified into two subgroups during our meta-analysis: chemo-sensitive and chemo-resistant. The data summary of the samples in our meta-analysis was shown in Table 2.

**Table 2:** Characteristics of the samples used in the meta-analysis.

Accession	Group	Study	Treatment	Platform	Tissue	Gender	Outcome	Age
GSM1278195	R.529	GSE52919	AraC/DNR	Agilent	MNC_Bone marrow	M	Resistance	43
GSM1278196	R.529	GSE52919	AraC/DNR	Agilent	MNC_Bone marrow	F	Resistance	61
GSM1278197	R.529	GSE52919	AraC/DNR	Agilent	MNC_Bone marrow	F	Resistance	32
GSM1278198	R.529	GSE52919	AraC/DNR	Agilent	MNC_Bone marrow	F	Resistance	43
GSM1278200	S.529	GSE52919	AraC/DNR	Agilent	MNC_Bone marrow	F	Sensitive	43
GSM1278201	S.529	GSE52919	AraC/DNR	Agilent	MNC_Bone marrow	M	Sensitive	50
GSM1278203	S.529	GSE52919	AraC/DNR	Agilent	MNC_Bone marrow	F	Sensitive	33
GSM1278204	S.529	GSE52919	AraC/DNR	Agilent	MNC_Bone marrow	F	Sensitive	44
GSM1278205	S.529	GSE52919	AraC/DNR	Agilent	MNC_Bone marrow	M	Sensitive	50
GSM1278206	S.529	GSE52919	AraC/DNR	Agilent	MNC_Bone marrow	F	Sensitive	18
GSM1278207	S.529	GSE52919	AraC/DNR	Agilent	MNC_Bone marrow	F	Sensitive	44
GSM1278208	S.529	GSE52919	AraC/DNR	Agilent	MNC_Bone marrow	M	Sensitive	44
GSM1277549	R.528	GSE52891	AraC + Anthra	HG-U133_Plus_2	Blast_Bone marrow_Blood	M	Resistance	Chilhood
GSM1277551	R.528	GSE52891	AraC + Anthra	HG-U133_Plus_2	Blast_Bone marrow_Blood	M	Resistance	Chilhood
GSM1277553	R.528	GSE52891	AraC + Anthra	HG-U133_Plus_2	Blast_Bone marrow_Blood	M	Resistance	Chilhood
GSM1277554	R.528	GSE52891	AraC + Anthra	HG-U133_Plus_2	Blast_Bone marrow_Blood	M	Resistance	Chilhood
GSM1277556	R.528	GSE52891	AraC + Anthra	HG-U133_Plus_2	Blast_Bone marrow_Blood	F	Resistance	Chilhood
GSM1277557	R.528	GSE52891	AraC + Anthra	HG-U133_Plus_2	Blast_Bone marrow_Blood	M	Resistance	Chilhood
GSM1277558	R.528	GSE52891	AraC + Anthra	HG-U133_Plus_2	Blast_Bone marrow_Blood	M	Resistance	Chilhood
GSM1277559	R.528	GSE52891	AraC + Anthra	HG-U133_Plus_2	Blast_Bone marrow_Blood	M	Resistance	Chilhood
GSM1277560	R.528	GSE52891	AraC + Anthra	HG-U133_Plus_2	Blast_Bone marrow_Blood	M	Resistance	Chilhood
GSM1277561	R.528	GSE52891	AraC + Anthra	HG-U133_Plus_2	Blast_Bone marrow_Blood	M	Resistance	Chilhood
GSM1277562	R.528	GSE52891	AraC + Anthra	HG-U133_Plus_2	Blast_Bone marrow_Blood	M	Resistance	Chilhood
GSM1277563	R.528	GSE52891	AraC + Anthra	HG-U133_Plus_2	Blast_Bone marrow_Blood	F	Resistance	Chilhood
GSM1277565	R.528	GSE52891	AraC + Anthra	HG-U133_Plus_2	Blast_Bone marrow_Blood	F	Resistance	Chilhood
GSM1277567	R.528	GSE52891	AraC + Anthra	HG-U133_Plus_2	Blast_Bone marrow_Blood	M	Resistance	Chilhood
GSM1277568	R.528	GSE52891	AraC + Anthra	HG-U133_Plus_2	Blast_Bone marrow_Blood	M	Resistance	Chilhood
GSM1277569	R.528	GSE52891	AraC + Anthra	HG-U133_Plus_2	Blast_Bone marrow_Blood	F	Resistance	Chilhood
GSM1277570	R.528	GSE52891	AraC + Anthra	HG-U133_Plus_2	Blast_Bone marrow_Blood	M	Resistance	Chilhood
GSM2867943	S.107	GSE107465	Anthra + nucleoside Analog	HG-U133_Plus_2	Blood	F	Sensitive	63
GSM2867944	S.107	GSE107465	Anthra + nucleoside Analog	HG-U133_Plus_2	Blood	F	Sensitive	54
GSM2867946	R.107	GSE107465	Anthra + nucleoside Analog	HG-U133_Plus_2	Blood	M	Resistance	69
GSM2867949	R.107	GSE107465	Anthra + nucleoside Analog	HG-U133_Plus_2	Blood	M	Resistance	76
GSM2867952	S.107	GSE107465	Anthra + nucleoside Analog	HG-U133_Plus_2	Blood	M	Sensitive	30
GSM2867954	R.107	GSE107465	Anthra + nucleoside Analog	HG-U133_Plus_2	Blood	F	Resistance	49
GSM2867955	S.107	GSE107465	Anthra + nucleoside Analog	HG-U133_Plus_2	Blood	M	Sensitive	53
GSM2867959	S.107	GSE107465	Anthra + nucleoside Analog	HG-U133_Plus_2	Blood	F	Sensitive	56
GSM2867965	S.107	GSE107465	Anthra + nucleoside Analog	HG-U133_Plus_2	Blood	M	Sensitive	32
GSM1099774	R.45	GSE45249	AraC + DNR	HG-U133A_2	LSC_Bone marrow	NA	Resistance	Childhood
GSM1099775	R.45	GSE45249	AraC + DNR	HG-U133A_2	LSC_Bone marrow	NA	Resistance	Childhood
GSM1099777	R.45	GSE45249	AraC + DNR	HG-U133A_2	LSC_Bone marrow	NA	Resistance	Childhood
GSM1099778	R.45	GSE45249	AraC + DNR	HG-U133A_2	LSC_Bone marrow	NA	Resistance	Childhood
GSM1099780	R.45	GSE45249	AraC + DNR	HG-U133A_2	LSC_Bone marrow	NA	Resistance	Childhood
GSM1099781	R.45	GSE45249	AraC + DNR	HG-U133A_2	LSC_Bone marrow	NA	Resistance	Childhood
GSM1099782	R.45	GSE45249	AraC + DNR	HG-U133A_2	LSC_Bone marrow	NA	Resistance	Childhood
GSM1099783	R.45	GSE45249	AraC + DNR	HG-U133A_2	LSC_Bone marrow	NA	Resistance	Childhood
GSM1099784	R.45	GSE45249	AraC + DNR	HG-U133A_2	LSC_Bone marrow	NA	Resistance	Childhood
GSM1099785	R.45	GSE45249	AraC + DNR	HG-U133A_2	LSC_Bone marrow	NA	Resistance	Childhood
GSM1099789	R.45	GSE45249	AraC + DNR	HG-U133A_2	LSC_Bone marrow	NA	Resistance	Childhood
GSM3265141	R.750	GSE75086	AraC	HuGene-2_0-st	Blast_Blood	NA	Resistance	NA
GSM3265143	R.750	GSE75086	AraC	HuGene-2_0-st	Blast_Blood	NA	Resistance	NA
GSM3265145	R.750	GSE75086	AraC	HuGene-2_0-st	Blast_Blood	NA	Resistance	NA
GSM3265147	R.750	GSE75086	AraC	HuGene-2_0-st	Blast_Blood	NA	Resistance	NA

*Anthra* Anthracycline, *DNR* Daunorubicin, *AraC* Cytarabine, *NA* not available.

### Quality assessment of the calibrated data

**Figure 1:**
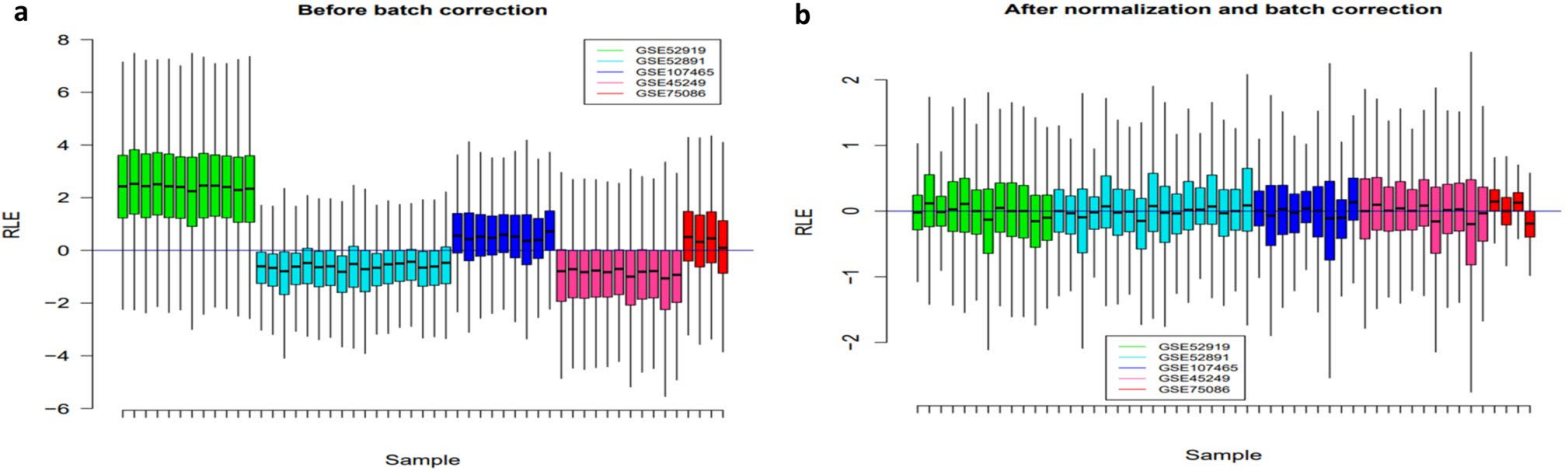
Boxplot for the RLE values. The relative log expression (RLE) box plot of AML sample arrays before (**a**) and after (**b**) batch correction. RLE distribution centered on zero demonstrates almost elimination of unwanted variation. The plot was generated using R language program version 4.0.5 (https://www.R-project.org/)^57^.

Before meta-analysis, probe annotation and filtering were applied on log-scaled features with identical distribution across all arrays, and genes with low variance in intensities across samples, control probe sets, and other internal controls were removed. To perform quality control assessment, RNA degradation plots and study-specific clustering pattern of samples were made. To reduce the batch effect, the well-established LIMMA (Linear Models for Microarray Data) procedure was applied and relative log expression (RLE) plots as a simple powerful tool for detecting and visualizing unwanted variations were used (Fig. 1a,b). As illustrated in Fig. 1b, in most cases, distribution of the chips was centered on about zero. No major differences to represent a bias were seen in our analysis.

**Figure 2:**
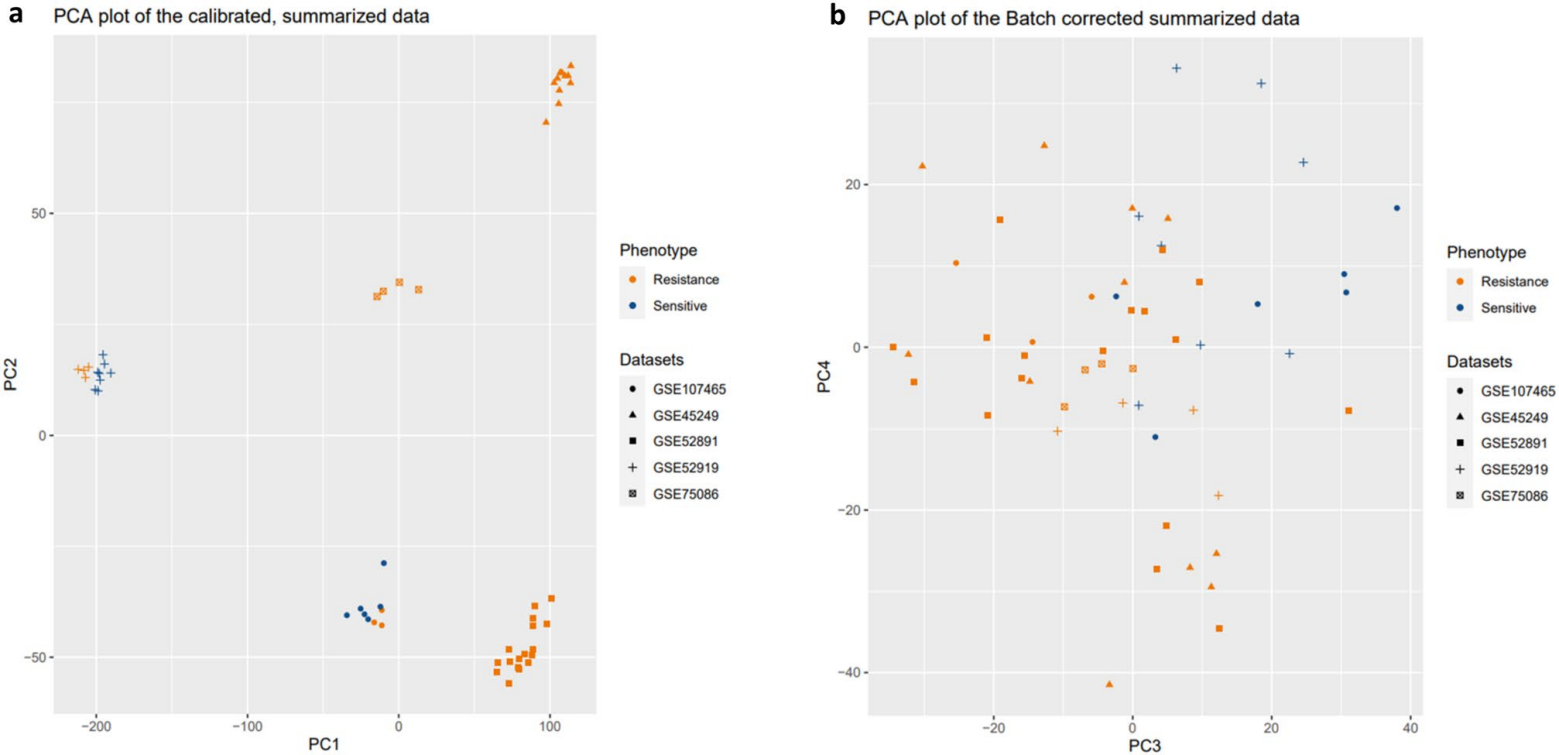
PCA plot of the summarized data by batch correction. Scatter plots of principal components analysis (PCA) show the PC1 versus PC2 output for each calibrated AML samples of included datasets before batch correction procedure (**a**), and PC3 versus PC2 output after batch correction (**b**). PCA plots illustrate the similarity of gene expression profiles among samples using the ggplot2 package version 3.3.3 in R^74^.

Moreover, to inspect the sample clustering patterns, the results were presented using the principal component analysis (PCA) plot which showed clustering based primarily on sensitive and resistant groups to chemotherapeutic AML drugs, PCA plot of the calibrated, summarized data, PC1/PC2 versus PCA plot of batch corrected summarized data, PC3/PC4 (Fig. 2a,b, and see Supplementary Fig. 1).

### Statistical meta-analysis

#### Identification of common gene expression signatures in chemo-resistance AMLs

**Figure 3:**
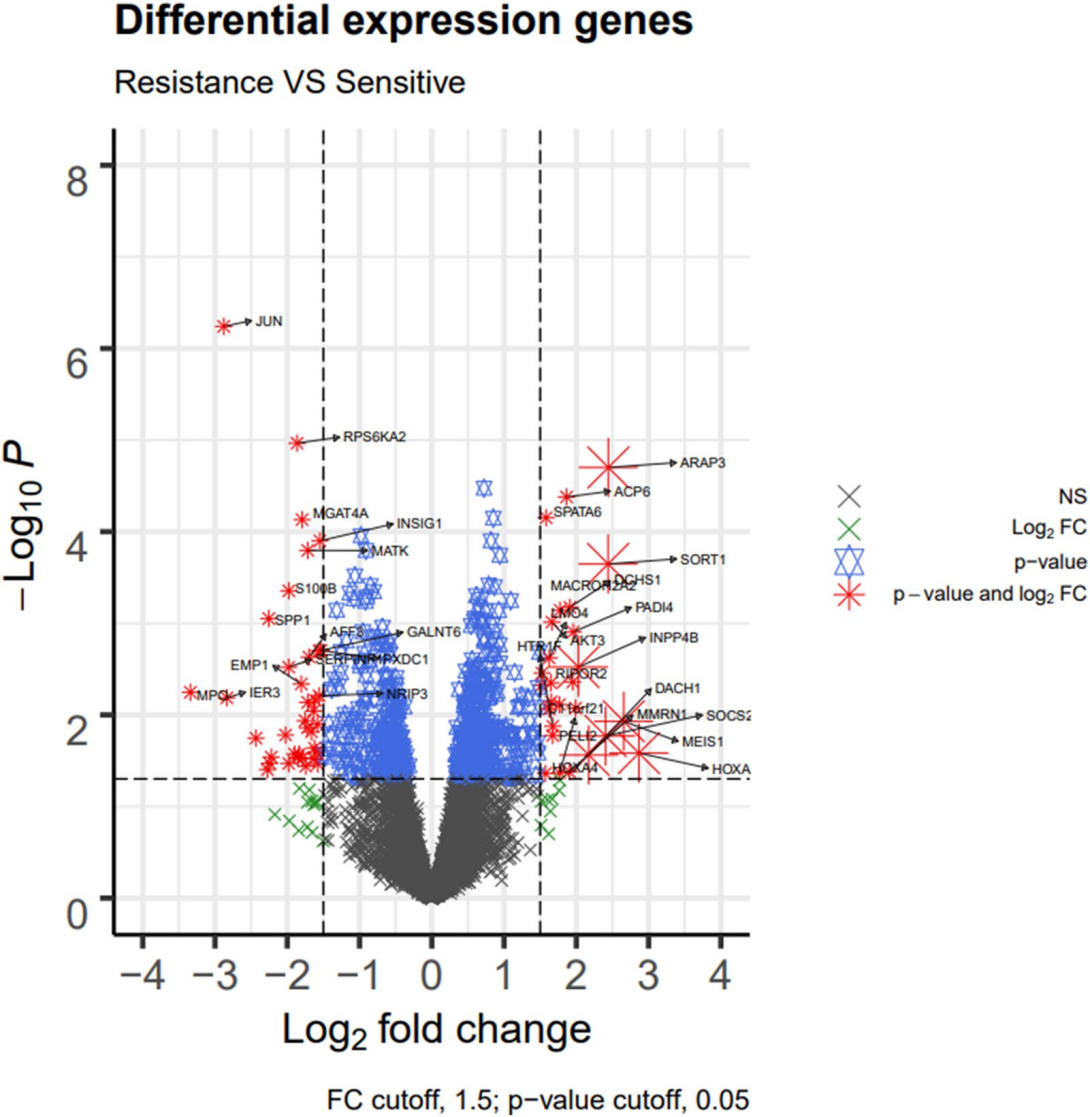
Volcano plot of the differentially expressed genes. Differentially expressed genes which were identified by meta-analysis were illustrated using the ggplot2 package in R^74^. *P* value cutoff was 0.05. As presented, 34 genes were identified as common differentially expressed genes (DEGs) with more than 1.5 fold change in chemo-resistance group as compared to the chemo-sensitive group. The data presented as log2 fold change. The plot was created by the ggplot2 R package version 3.3.3^63^. *FC* fold change, *NS* not significant.

**Figure 4:**
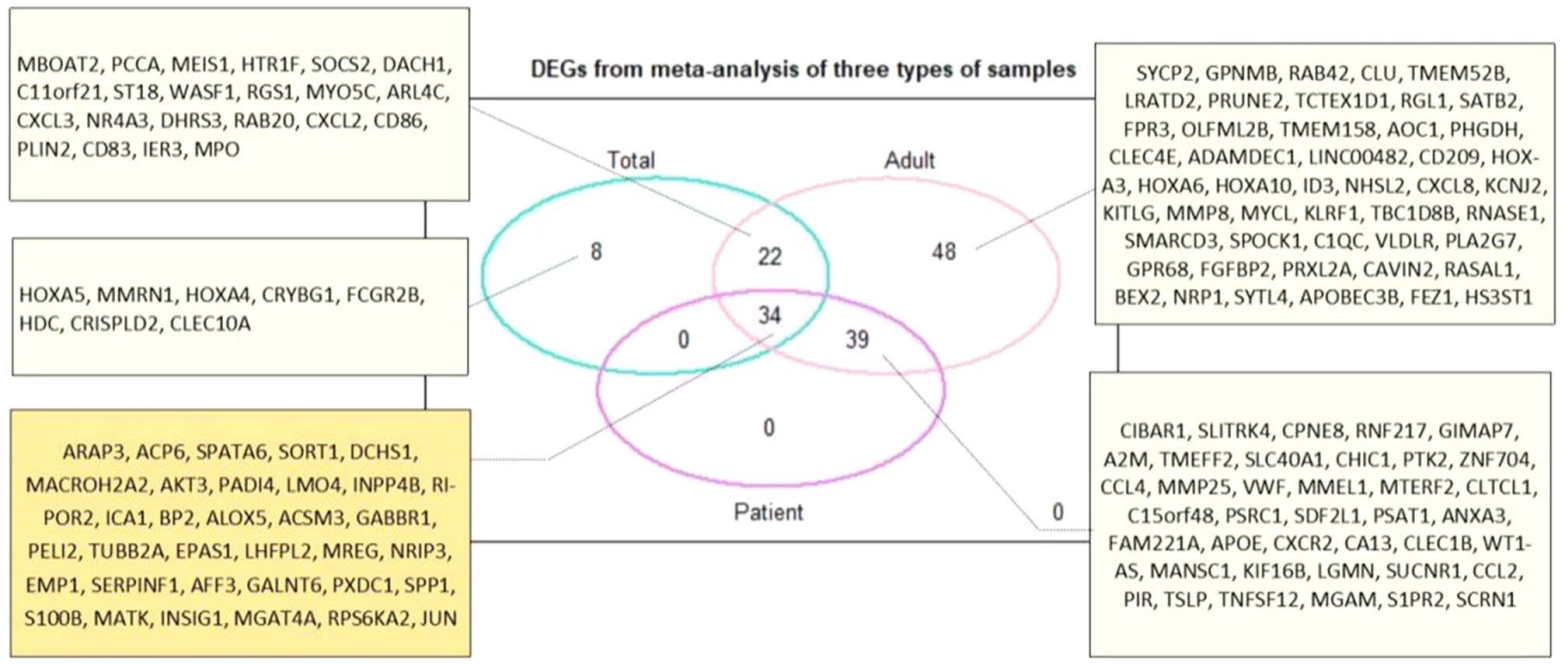
Venn diagram of DEGs. It shows the results of 3 statistical meta-analysis with difference in included sample arrays from all types of sample in total, only patient’s samples, and only adult patient’s samples. 34 genes were identified as common significantly differentially expressed genes (DEGs) between the resistance versus sensitive among all type of analysis (genes with a *P* value < 0.05 and |fold change|> 1.5 were considered significant). The plot was created using the Package limma version 3.44.3 in R^63^.

After normalization of all datasets, integration analysis was performed in three steps: (1) Analysis of all normalized arrays including patient’s cells (subpopulations of LSC) and patient’s samples from adult and childhood patients (53 sample arrays). (2) Analysis of arrays from patients’ samples (38 sample arrays). (3) Analysis of adult patients (21 sample arrays). Using LIMMA approach and considering the cut-off criteria of *P *value < 0.05 and |fold change|> 1.5 (Fig. 3), a total of 64, 73 and 143 DEGs were found in chemo-resistant group compared to sensitive group for each mentioned analysis, respectively (Fig. 4). The top significantly up- or down-regulated genes in the first meta-analysis were shown in Fig. 3.

**Figure 5:**
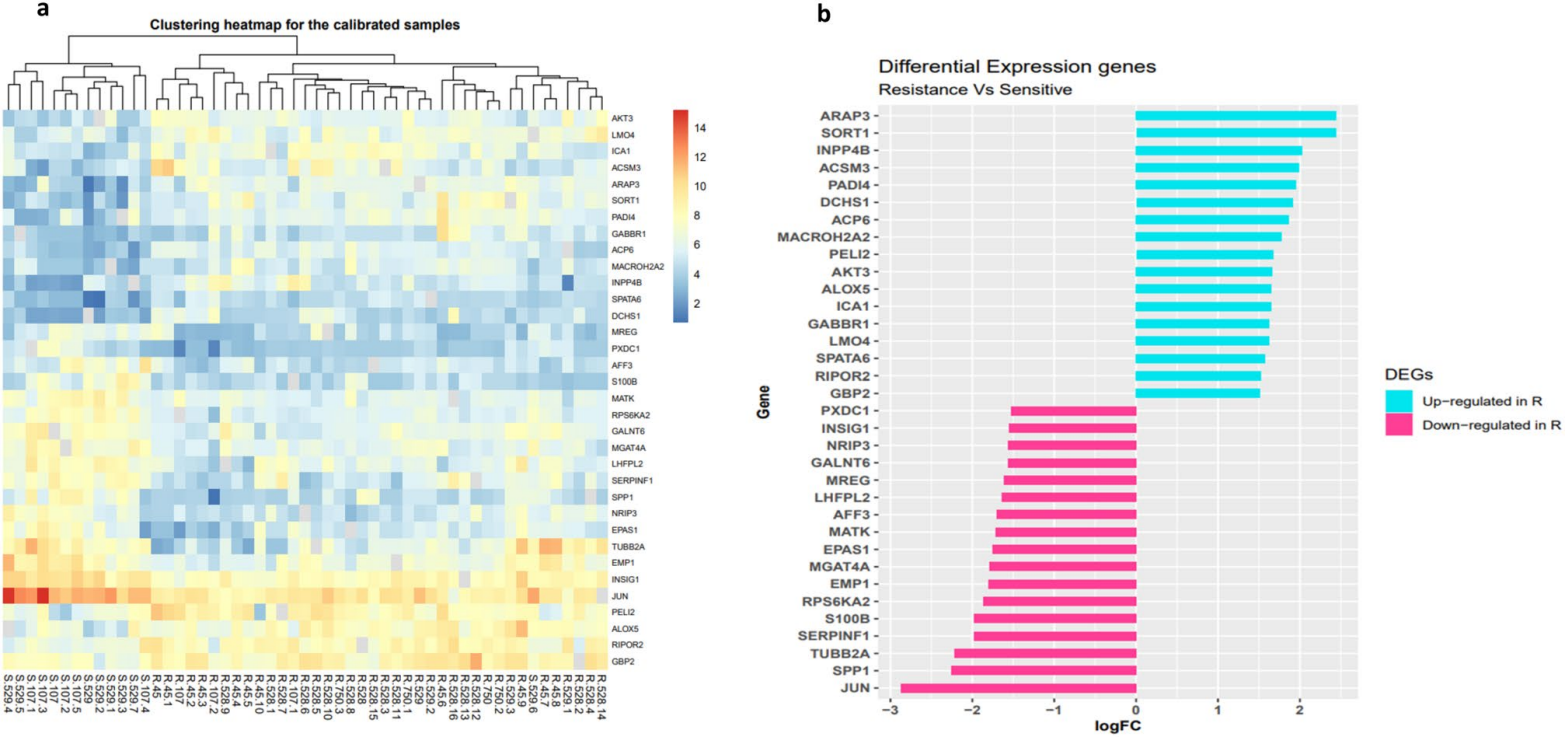
Heatmap and bar plot of Log fold changes of the most significantly DEGs. (**a**) The heatmap indicates the normalized relative expression value of the 34 most significantly differentially expressed genes (DEGs) between AML chemo-resistance and AML chemo-sensitive samples. Each column represents AML samples and all samples were divided to two clusters based on their characteristic (chemo-resistant and chemo-sensitive) by a hierarchical clustering analysis. Hierarchical clustering for the scaled gene expression matrix was based on the Euclidean correlation. The gradual color from orange to blue represents the expression changes from upregulated to downregulated genes. (**b**) The plot shows fold change of differential expression genes including 17 downregulated (pink) and 17 upregulated (blue) statuses in resistance group in compared to sensitive group. The Figure was created using the ggplot2 package version 3.3.3 in R^74^.

Among 64 DEGs which were significantly identified in the first analysis, 34 annotated genes were common with two other statistical analyses like *c-Jun* (also known as *JUN*), *ARAP3, SORT1*, *SPP1*, *RPS6KA2*, *ACP6, AKT3, PELI2, GABBR1, MATK* genes (for more details please see Fig. 4 and Table 3). In other word, these 34 differentially expressed genes where 17 genes were up-regulated and 17 genes were down-regulated in AML chemo-resistance group compared to chemo-sensitive group, have a possible role in development of drug resistance in general in AML adult patient. Among DEGs, *c-Jun* was identified as the most significant deregulated genes with the considerable decreased expression (with fold change, − 2.87 and *P* value, 5.74E−07) in the chemo-resistant AML samples. In contrast, the data showed significantly increased expression of *ARAP3* and *SORT1* genes (with logFC, 2.4 and *P* value < 0.01). The heatmap graph of the most differentially regulated genes with possible role in AML drug resistance and their corresponding fold changes were shown in Fig. 5.

**Table 3:** Identified differentially expressed genes (DEGs) associated with AML drug resistance.

ID	Gene symbol	logFC	*P* value
64411	ARAP3	2.442523	1.99E−05
6272	SORT1	2.439522	0.000225
8821	INPP4B	2.028562	0.003002
6296	ACSM3	1.989434	0.008442
23569	PADI4	1.95709	0.001243
8642	DCHS1	1.911221	0.000669
51205	ACP6	1.86439	4.18E−05
55506	MACROH2A2	1.775521	0.000722
57161	PELI2	1.672159	0.016775
10000	AKT3	1.661464	0.00097
240	ALOX5	1.652833	0.007188
3382	ICA1	1.64897	0.004476
2550	GABBR1	1.623574	0.008618
8543	LMO4	1.622589	0.002394
54558	SPATA6	1.581695	7.04E−05
9750	RIPOR2	1.526171	0.00349
2634	GBP2	1.516136	0.004751
221749	PXDC1	− 1.53333	0.001975
3638	INSIG1	− 1.54813	0.000126
56675	NRIP3	− 1.55975	0.006188
11226	GALNT6	− 1.56183	0.002004
55686	MREG	− 1.61191	0.006915
10184	LHFPL2	− 1.63614	0.009076
3899	AFF3	− 1.69853	0.002341
4145	MATK	− 1.71664	0.00016
2034	EPAS1	− 1.75365	0.011568
11320	MGAT4A	− 1.79541	7.39E−05
2012	EMP1	− 1.80654	0.004605
6196	RPS6KA2	− 1.86465	1.08E−05
6285	S100B	− 1.97875	0.000442
5176	SERPINF1	− 1.98061	0.003015
7280	TUBB2A	− 2.22182	0.028914
6696	SPP1	− 2.25565	0.000888
3725	JUN	− 2.87946	5.74E−07

#### ROC curve analysis

**Figure 6:**
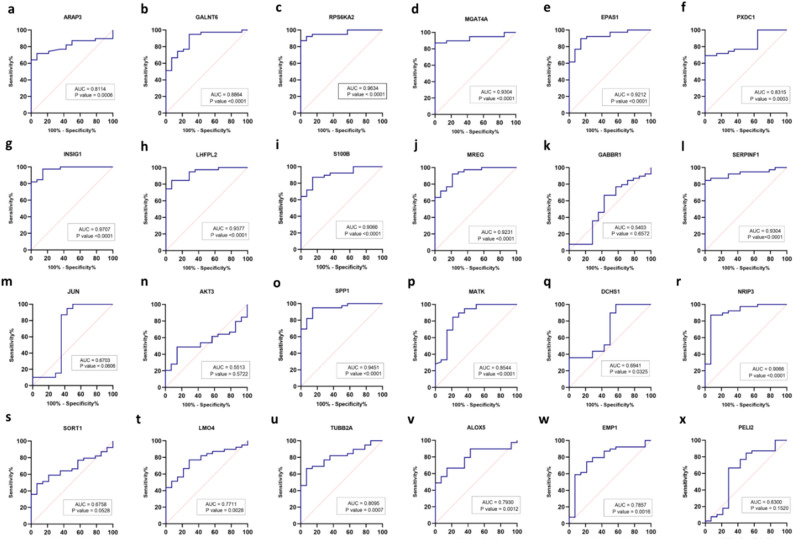
ROC curve analysis for DEGs. Receiver operating characteristics (ROC) curve was constructed and performed using GraphPad.Prism.9 (https://www.graphpad.com) to assess the predictive accuracy of gene signature for AML chemo-resistance. The AUC value was 0.7 demonstrating predictive power of the identified genes.

The ROC curve analysis was performed by the GraphPad.Prism.9 software. As shown in Fig. 6, the area under the curve (AUC) of most of the identified genes was above 0.7 (AUC > 0.7). Among these hub genes, AUC for *RPS6KA2*, *S100B*, *INSIG1, EPAS1, MGAT4A, NRIP3, SERPINF1, SPP1, LHFPL2* and *MERG,* was 0.9 (AUC ≥ 0.9). This indicated that these genes could be considered as valuable predictive biomarkers for chemo-resistance onset in AML. The AUCs of other DEGs were less than 0.7 (Fig. 6).

#### Functional gene enrichment analysis

**Figure 7:**
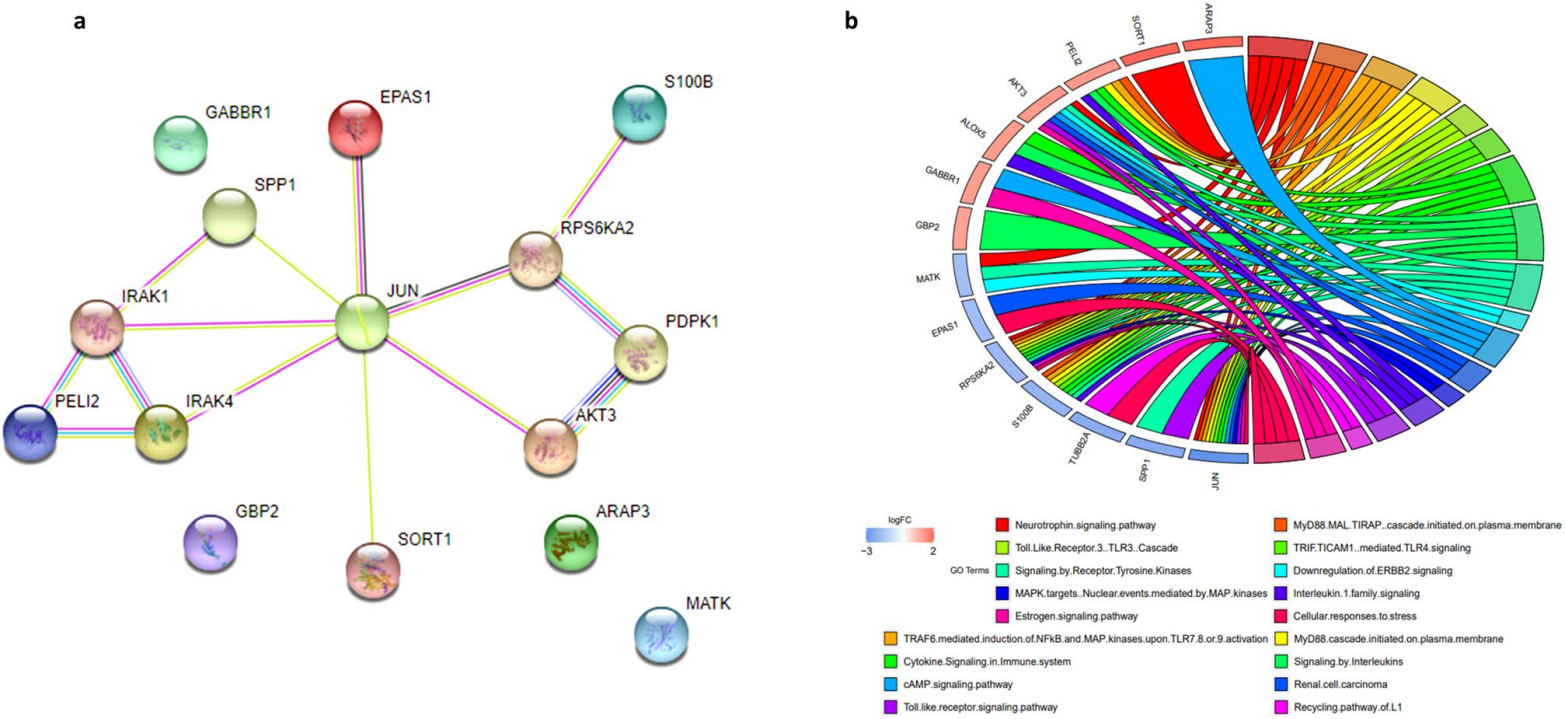
Pathway enrichment among AML deregulated genes associated with chemotherapeutic resistance. (**a**) Protein–protein interaction (PPI) network of dysregulated genes with more significance based on KEGG biological pathways. The protein–protein association network was retrieved from the STRING enrichment web service (https://string-db.org/) using the StringApp in the Cytoscape v3.7.0 (https://cytoscape.org/) and additional interactors were added. (**b**) Circos diagram depicts KEGG (https://www.kegg.jp/kegg/kegg1.html) and Reactome (https://reactome.org/) signaling pathways enriched for DEGs with probable contribution to chemotherapy resistance. The Circos diagram was generated using the ggplot2 package version 3.3.3 in R^74^.

To clarify the biological roles of DEGs in development of AML drug resistance, functional gene enrichment analysis was conducted using the Enrichr for 34 genes which were identified as common DEGs in three statistical analyses (see Fig. 7). Enrichr provided gene ontology (GO) enrichment including the biological process (BP), molecular function (MF) and cellular component (CC) categories as well as web-based pathway analysis to map genes to pathways created by Kyoto Encyclopedia of Genes and Genomes (KEGG) and Reactome online resources^12–14^.

For gene set enrichment we also used the functional annotation chart tool of DAVID (Table 4) and StringApp plug-in implemented in Cytoscape v.3.7.0 (Table 5 and Supplementary Fig. 2). Finally, common terms with cut-off *P* value < 0.05 including “neurotrophin signaling pathway”, “cAMP signaling pathway”, “Toll-like receptor signaling pathway”, “Renal cell carcinoma” and “Estrogen signaling pathway” were identified as the most strongly enriched pathway clusters for DEGs using all procedures with potential responsibility in AML chemo-resistance based on KEGG (Table 4). Among DEGs both *c-Jun* and *AKT3* genes were involved in all of the five identified pathways with − 2.88 and 1.66 logFC, respectively, as well as *P* value < 0.01. Moreover, neurotrophin signaling pathway was the most highly enriched pathway associated with AML drug resistance with the five deregulated mediators including the upregulation of *SORT1* and *AKT3* genes, and the downregulation of *c-Jun, MATK*, and *RPS6KA2* genes. The pathway was indicated by DAVID (*P* value, 1.04E−04), StringApp (FDR value, 2.60E−04) and Enrichr (*P* value, 1.66E−06 and Combined Score, 399.566) as the most significantly over-represented signal transduction for driving drug resistance in AML (Tables 4, 5).

**Table 4:** Functional annotation using chart tool of DAVID.

Category	Term	*P* value	Fold enrichment
KEGG_PATHWAY	hsa04722:Neurotrophin signaling pathway	1.04E−04	17.91406
GOTERM_CC_DIRECT	GO:0048471~perinuclear region of cytoplasm	0.003684	5.502415
GOTERM_BP_DIRECT	GO:0008283~cell proliferation	0.004387	7.168716
KEGG_PATHWAY	hsa04024:cAMP signaling pathway	0.008276	8.685606
KEGG_PATHWAY	hsa05211:Renal cell carcinoma	0.008783	19.54261
UP_KEYWORDS	Polymorphism	0.012066	1.357115
GOTERM_CC_DIRECT	GO:0005829~cytosol	0.016845	2.061538
KEGG_PATHWAY	hsa04915:Estrogen signaling pathway	0.019058	13.02841
KEGG_PATHWAY	hsa04620:Toll-like receptor signaling pathway	0.021672	12.16804
GOTERM_MF_DIRECT	GO:0005515~protein binding	0.025396	1.381129
UP_SEQ_FEATURE	Sequence variant	0.031799	1.280429
UP_KEYWORDS	Golgi apparatus	0.039739	3.727362
GOTERM_CC_DIRECT	GO:0005667~transcription factor complex	0.042441	8.852332

**Table 5:** Pathways and genes identified using StringApp (related to Fig. 7b).

Category	Description	FDR value	Genes
KEGG pathways	Neurotrophin signaling pathway	2.60E−04	SORT1|AKT3|JUN|MATK|RPS6KA2
Reactome pathways	MyD88:MAL(TIRAP) cascade initiated on plasma membrane	0.0041	PELI2|S100B|JUN|RPS6KA2
Reactome pathways	TRAF6 mediated induction of NFkB and MAP kinases upon TLR7/8 or 9 activation	0.0041	PELI2|S100B|JUN|RPS6KA2
Reactome pathways	MyD88 cascade initiated on plasma membrane	0.0041	PELI2|S100B|JUN|RPS6KA2
Reactome pathways	Toll Like Receptor 3 (TLR3) Cascade	0.012	S100B|JUN|RPS6KA2
Reactome pathways	TRIF(TICAM1)-mediated TLR4 signaling	0.012	S100B|JUN|RPS6KA2
Reactome pathways	Cytokine Signaling in Immune system	0.0131	PELI2|S100B|GBP2|JUN|ALOX5|RPS6KA2
Reactome pathways	Signaling by Interleukins	0.0134	PELI2|S100B|JUN|ALOX5|RPS6KA2
Reactome pathways	Signaling by Receptor Tyrosine Kinases	0.0134	AKT3|S100B|MATK|SPP1|RPS6KA2
Reactome pathways	Downregulation of ERBB2 signaling	0.015	AKT3|MATK
KEGG pathways	cAMP signaling pathway	0.0162	ARAP3|AKT3|JUN|GABBR1
KEGG pathways	Renal cell carcinoma	0.0162	EPAS1|AKT3|JUN
Reactome pathways	MAPK targets/ Nuclear events mediated by MAP kinases	0.0185	JUN|RPS6KA2
Reactome pathways	Interleukin-1 family signaling	0.0202	PELI2|S100B|ALOX5
KEGG pathways	Toll-like receptor signaling pathway	0.0256	AKT3|JUN|SPP1
Reactome pathways	Recycling pathway of L1	0.034	TUBB2A|RPS6KA2
KEGG pathways	Estrogen signaling pathway	0.0431	AKT3|JUN|GABBR1
Reactome pathways	Cellular responses to stress	0.0456	EPAS1|JUN|TUBB2A|RPS6KA2

*FDR* false discovery rate.

## Discussion

In this study we wished to investigate deregulated genes and enriched pathways involved in drug resistance in AML patients under treatment of DNA-damaging agents including Anthracyclines, Cytarabine and Gemtuzumab ozogamicin. Analysis of transcriptomic profiles of AML samples was performed on two groups, chemo-resistance against chemo-sensitive. Moreover, a comparative meta-analysis in three ways was conducted based on sample type (only for patient’s sample not patient’s cells), age (only adult; due to unavailability of sensitive sample in childhood group) and samples in total (with any criteria). The data resulted in the identification of 34 common DEGs that were statistically correlated with AML chemo-resistance. In the next step, gene set enrichment analysis using DAVID/Enrichr/Cytoscape (StringApp) was done to identify possible signaling pathways which were enriched among candidate DEGs, and could be associated with chemo-refractory relapse to DNA-damaging compounds in AML patients after chemotherapy.

**Figure 8:**
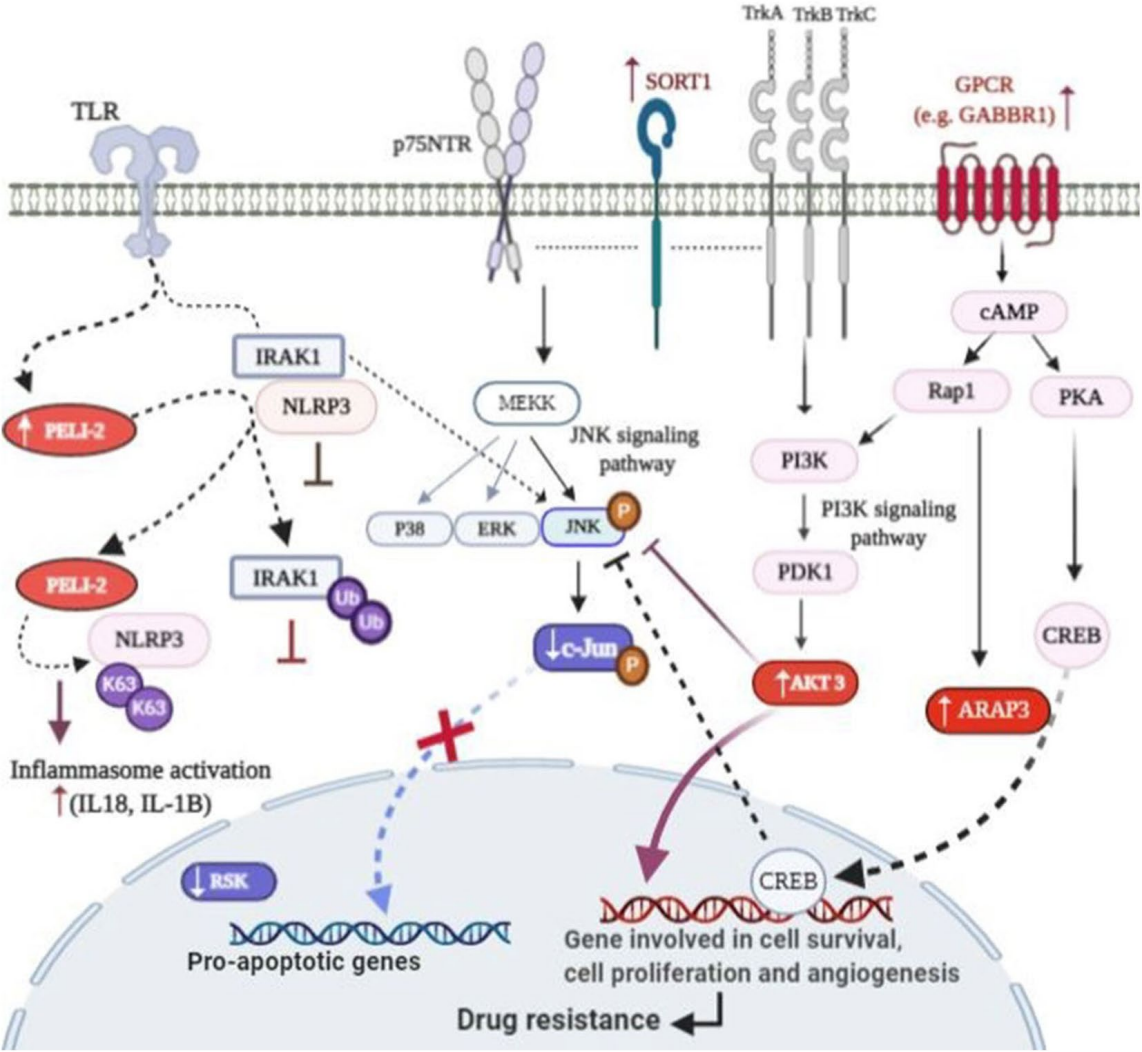
A schematic interplay of signaling pathways that influence/associate with AML drug resistance. The various molecular signaling pathways may very intricately involve in development of chemo-resistance including: neurotrophin signaling pathway, cAMP signaling pathway, TLR signaling pathway and estrogen receptor signaling pathways. *JUN*, *AKT3*, *ARAP3, SORT1, GABBR1,* and *PELI2* are deregulated genes contributed to AML drug resistance. In AML cells under treatment, concurrent up-regulation of *AKT3* and down-regulation of *Jun*, downstream of all pathways, suppress apoptosis-induced JNK. JNK signaling is one of the deaths responses downstream of these pathways. Therefore, failure in JNK activation could be one of the main causes of chemo-resistance in AML. Up-regulation of *AKT3* in PI3K/AKT signaling cascade induces expression of genes involved in cell survival, cell proliferation and angiogenesis. Also, down-regulation of Jun leads to decreased expression of pro-apoptotic genes. Participation of ARAP3 as well as AKT3 in angiogenesis can intensify resistance to chemotherapy. In neurotrophin signaling pathway, the high contribution of SORT1 with Trk receptors may have an oncogenic effect for AML cells and can promote cell survival. Downstream of TLR signaling pathway, PELI2 can activate inflammasome complex through concurrent of ubiquitination of two parallel targets, NLRP3 and IRAK1. The IRAK1 inactivation leads to the release of IL-1β and IL-18 through activation of inflammasome. IL-1β and IL-18 activation may be a distinct plausible important mechanism by which PELI2 was involved in emergence of drug resistance. The Figure was created using BioRender (https://biorender.com/).

Our data revealed the involvement of five major signaling pathways associated with chemo-refractory relapse in AML samples. These signaling pathways were as follow: neurotrophin, Estrogen, cAMP, Toll-like receptor and Renal cell carcinoma. Among the above pathways, neurotrophin signaling pathway was found to be the most significantly over-represented signal transduction for driving drug resistance in AML as indicated in Tables 4 and 5. Its tyrosine kinase receptors [tropomyosin receptor kinase (Trk)] including Trk A, B and C, express in a variety of human tissues and support cell survival in multiple solid and liquid tumors^15,16^. Moreover, neurotrophin/Trk signaling pathway has been found connected with a variety of intracellular cascades including mitogen-activated protein kinase (MAPK) pathway, the phosphatidylinositol 3-kinase (PI3K)/AKT pathway, and phospholipase C (PLC) pathway, providing growth and survival advantage for cells^17,18^. On the contrary, the p75 neurotrophin receptor (p75NTR), another receptor of neurotrophins, induces the expression of pro-apoptotic genes through activation of p38 and c-Jun N-terminal kinase (JNK) pathways^18^ (see Fig. 8).

Our meta-analysis data revealed that the expression of *AKT3*, *RPS6KA2*, *c-Jun*, *SORT1* and *MATK* genes was significantly altered in chemo-resistance AML samples. *SORT1, AKT3* and *RPS6KA2* genes were up-regulated but *c-Jun* and *MATK* were downregulated in AML chemo-resistance group compared to the -sensitive group (see Fig. 5b)*.* It has been reported that AKT3 has an important role in DNA double strand break repair and chemotherapeutic resistance^20^. It has been documented that both *AKT3* and *RPS6KA2* genes could act in parallel as the mediators of PI3K/AKT and MAPK pathways under the impression of neurotrophin signals^15,21^. c-Jun has been reported to play an apoptotic role in neurotrophin/Trk signaling pathway^18^.

The contribution of SORT1 (also known as *sortilin*) with Trk receptors promotes cell survival and characterized as an oncogenic factor for cells^18^. This gene was the second significantly up-regulated gene in chemo-resistant AML samples, which may induce resistance to chemotherapy through neurotrophin signaling pathway. It was reported that the expression of *SOTR1* was elevated in adult Acute B Lymphoblastic Leukemia (B-ALL) cases after chemotherapy, which was correlated to relapse and/or B-ALL-related death^22^.

cAMP signaling pathway is the second pathway that was identified in our chemo-resistance AML samples. This pathway is one of the important cascades associated with anthracycline resistance in AML patients^23^. It has been demonstrated that cAMP plays a crucial role in the reduction of response to DNA-damaging reagents in Chronic myelogenous leukemia (CML) cells^23^. Moreover, it has been shown that cAMP signaling pathway is under regulation of G protein-coupled receptors (GPCRs) which contribute to the development of AML^24^. Besides, the elevation of cAMP signaling could suppress apoptosis-induced JNK activation^25^. Our meta-analysis data showed that *GABBR1*, a member of GPCR family, had an increased expression in chemo-resistance samples compared to the sensitive ones (logFC 1.6) (Fig. 5b)^26^. GABBR1 has been introduced as a survival associated marker for AML^27^. Excessive signal transduction through GABBR1 triggers growth and migration of cancer cells^27^. These findings support our data showing the association of increased expression of *GABBR1* and chemo-resistance in AML samples.

In addition to *GABBR1*, the expression level of other cAMP-related genes including *ARAP3*, *AKT3* were increased, but *c-Jun* was down regulated. ARAP3 and AKT3 are two down-stream elements of GPCRs oncogenic pathway. The critical role of ARAP3 and AKT isoforms was shown in regulating the developmental angiogenesis. These two proteins are common substrate for PI3K pathways which play an essential role in angiogenesis^28,29^. The importance of angiogenesis in AML as a source of drug resistance and relapse was documented in several clinical studies^30,31^.

Given the highest expression of *ARAP3* gene among 34 DEGs in our chemo-resistance samples, it could be suggested that increased level of ARAP3 may correlate with increased angiogenesis through PI3K pathway. This could also provide an explanation for the development of chemo-resistance response of AML patients to treatment. Therefore, *ARAP3* gene could be introduced as a high-risk marker in AML relapse, and could be considered as a new target for AML therapy. Analysis of receiver operating characteristic (ROC) curve, showed a significant area under the curve (AUC, 0.8114; *P *value < 0.01) (Fig. 6a), which further supported the importance of ARAP3 as a potential biomarker associated with chemo-resistance in AML.

Furthermore, many reports have confirmed the important role of Akt-related pathway in the development of resistance against DNA-damaging drugs in tumor cells^11,19,20,32,33^. It has been shown that Akt-related pathway could lead to resume DNA replication by recovery of genome stability, and drive cancer cells to M phase through stimulate expression of CDKN1A (cyclin-dependent kinase inhibitor 1A). Moreover, it has been shown that the increased *AKT3* gene expression could promote tumor malignancy and resistance to DNA-damaging chemotherapy compounds through activation of DNA repair pathway in glioma tumor cells^20,34^.

The next signaling pathway which was deregulated in our chemo-resistance AML samples was the estrogen signaling pathway. In this pathway, *AKT3*, *c-Jun* and *GABBR1* genes were deregulated in chemo-resistance AML samples, of which *AKT3* and *GABBR1* were upregulated, but *c-Jun* was down regulated. Recently, a preclinical study has considered estrogen receptors as a potential target to enhance chemotherapy for patients with AML^35^. Given the increased expression of *AKT3* and *GABBR1* in chemo-resistance AML samples, as shown in our meta-analysis data, it could be suggested that the estrogen signaling pathway might play an important role in the protection of leukemic cells from apoptosis.

Toll like receptor (TLR) signaling pathway was another signaling pathway in our gene set enrichment analysis data, which was correlated with chemo-resistance behavior of AML cells. It has been reported that Anthracyclines, as the immune-stimulatory chemotherapeutic agent, can promote TLRs-mediated immunogenic apoptotic cell death through increased emission of DAMPs by damaging DNA in tumor cells^36^. Based on our gene set enrichment analysis (GSEA) results, differential expression of several genes including *PELI2*, *RPS6KA2*, *S100B*, *c-Jun*, *AKT3*, and *SPP1*, may contribute to the aberrant signal transduction of TLRs upon TRIF/MyD88- mediated induction signaling in chemo-resistance AML group (Fig. 8).

In association with TLR pathway, our data showed an enhanced expression of *PELI2* gene in chemo-resistance group. *PELI2* (also known as *Pellino2*) encodes one of the members of the E3 ubiquitin ligases which regulate activation of NFκB (nuclear factor kappa enhancer binding protein) and MAPK cascades downstream of TLR signaling pathway. Studies on PELI2 have shown a reciprocal regulating interplay between PELI2 and IRAK1^37–39^. PELI2 interacts with IRAK1 and can be a kinase substrate of IRAK1^40^. In addition, it efficiently mediates polyubiquitination of IRAK1 in both Lys-63 and Lys-48 and induces TAK1-dependent JNK and ERK (Extracellular signal-regulated kinase) activation^38–40^. However, it appears that PELI2 involves in various cascades with a cell-type specific manner^41^. Recently, it was reported that PELI2 has a positive role in regulation of signaling-mediated NLRP3 inflammasome and increase caspase1-mediated activation of two immunoregulatory cytokines from IL-1 family, IL-1β and IL-18, in post-translational stage. PELI2 activates inflammasome complex through concurrent of ubiquitination of two parallel targets, NLRP3 and IRAK1. The inactivation of IRAK1 could suppress the activation of inflammasome^38^. Based on observations that IL-1β and IL-18 can contribute to AML anti-cancer drug resistance, and based on our data showing enhanced expression of *PELI2* gene, mediating IL-1β and IL-18 activation may be a distinct plausible important mechanism by which PELI2 involved in emergence of drug resistance^42–45^. In spite of this, some studies highlighted the central role of dysregulated IRAK1 and IRAK4 signaling in chemotherapy resistance^46^. Understanding the relationship between deregulated expression of *PELI2* gene and AML chemotherapy failure remains a challenge and further in vitro studies can provide important clues for its potential therapeutic usefulness.

The second gene in TLR signaling pathway was *RPS6KA2* (also known as *RSK3*) which belongs to the ribosomal S6 kinase family. Our data showed a reduced expression of *RPS6KA2* in chemo-resistant AML group. A previous study reported that RPS6KA2 was activated in vitro by c-Jun N-terminal kinase (JNK). Moreover, ROC curve analysis revealed the importance of *RPS6KA2* as a biomarker for AML chemo-resistance (AUC, 0.9634, *P *value < 0.0001) (Fig. 6c).

JNK signaling pathway has been shown to be activated by multiple receptors including GPCRs, TLRs, neurotrophin receptors (Trks), and estrogen receptors (ER), which generally promotes cell death and apoptosis through activation of c-Jun, an important pro-apoptotic protein, and inhibition of Akt-inducing survival signaling^47–50^. Interestingly, as illustrated in Fig. 8, consistent with the above facts, our meta- and gene set enrichment analysis in AML chemo-resistance samples showed significant inhibition of JNK signaling due to simultaneous down-regulation of *c-Jun* and up-regulation of *AKT3* expression.

Several studies have highlighted the critical role of JNK in Anthracycline induced apoptosis in AML cells^51,52^. These studies hypothesized that failure in JNK activation could be one of the main cause of resistance of AML cells to Anthracycline-containing treatment protocols^51,52^. These reports can further support our in silico findings that overexpression of *AKT3* and down-regulation of *c-Jun* could function as one of the main molecular mechanism for resistance of AML patients to chemotherapeutic protocols. Moreover, recently the importance of Akt inhibitors to improve the efficacy of DNA-targeting drugs has been suggested^32^. Therefore, we can suggest that reduced impact of JNK signaling at the intersection between multiple signaling pathways in AML cell may strengthen survival signaling in these cells against DNA-damaging drugs, resulting in chemo-resistance phenotype.

Furthermore, following down regulation of *c-Jun* expression, the expression of some multidrug efflux transporters (MDR) such as *ABCB1* might be decreased as well^53–55^. Supporting this notion, in the present meta-analysis no overexpression of these MDR genes observed in chemo-resistance samples (supplementary Table S1).

## Methods

### Dataset selection strategy

In order to retrieve related AML drug resistance published array expression datasets, the Gene Expression Omnibus (GEO) (https://www.ncbi.nlm.nih.gov/gds) and the ArrayExpress (https://www.ebi.ac.uk/arrayexpress) repositories were investigated. The search query included “AML” and “resistance/refractory/relapse” and “cytarabine (or Ara-C)” and “anthracycline”. The filters were “Homo sapiens” and “Expression profiling by array”. After removing duplicates and irrelevant datasets based upon inclusion of different criteria, such as tissue (bone marrow or peripheral blood), treatment (at least one course of induction chemotherapy with DNA-targeting drugs like anthracycline and cytarabine regimen) and platforms of microarray experiments, five microarray datasets with two different platforms including (Affymetrix and Agilent single channel arrays) were considered. Moreover, studies with unavailable raw data and poor quality were excluded.

The chemotherapeutic regimen considered in this study was composed of cytarabine, either alone or in combination with anthracyclines and other anti-cancer agents with DNA-damaging effect such as gemtuzumab ozogamicin. According to the outcome of the treatment with the above drugs, samples were classified into two groups: chemo-sensitive (complete remission after initial therapy) and chemo-resistance (relapse or refractory). The data included in this study were from AML patient’s samples as well as patient’s cells with blast or leukemic stem cell irrespective of their origin (bone marrow or peripheral blood).

**Figure 9:**
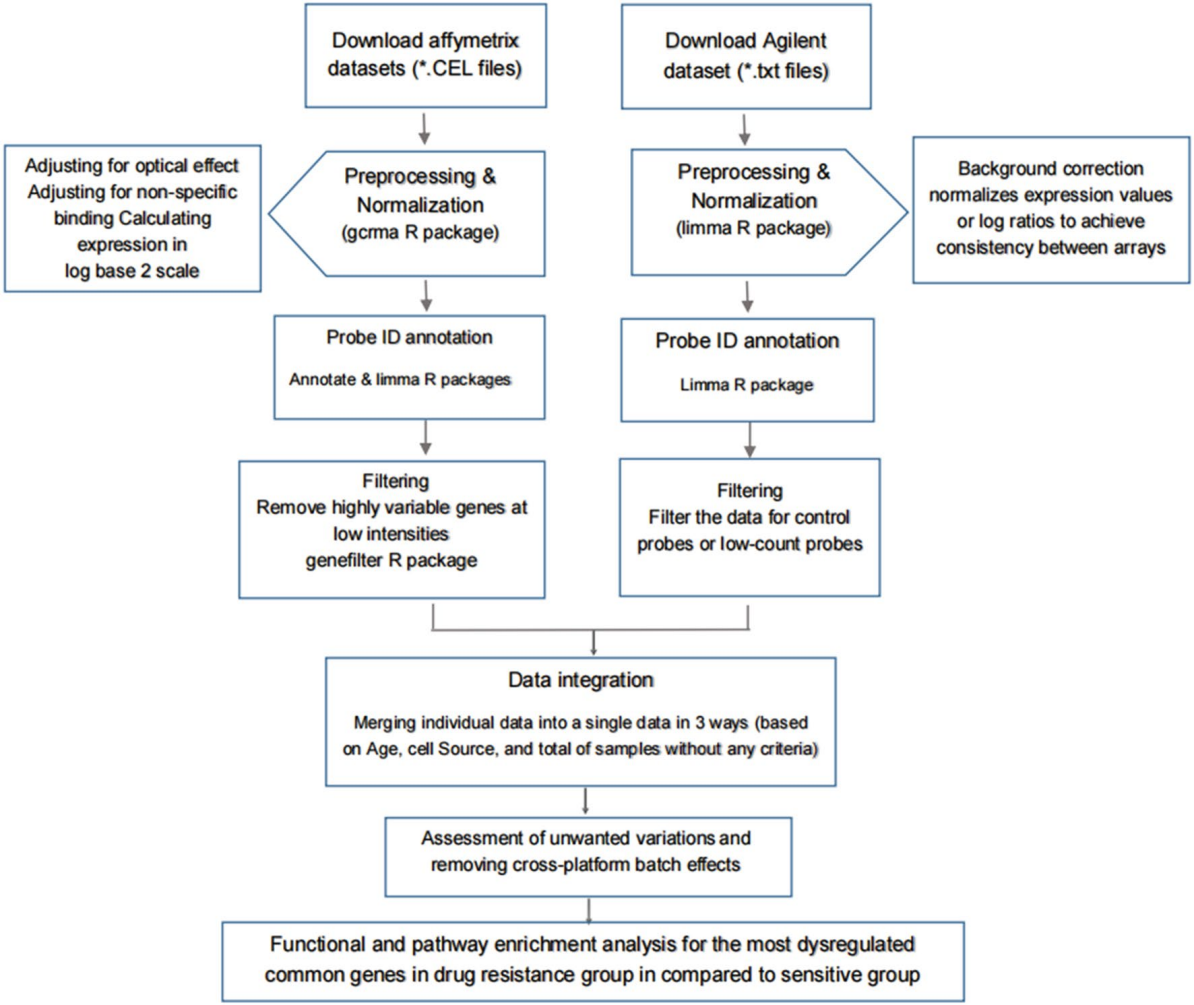
Workflow of conducting meta-analysis of microarray datasets.

Meta-analysis was first implemented on all five datasets regardless of age and sample source. Then samples were analyzed based on sample source (only patient’s samples not patient’s cells) as well as the patient’s age (only samples from adult patients were used). In each condition, separate cross integrative analysis was performed and gene set enrichment analysis was fulfilled for common DEGs. The overall workflow of the study design is illustrated in Fig. 9.

### Microarray data processing

The process of microarray data analysis including raw data quality control (QC), data pre-processing, assessing the effects of normalization, individual data annotation and analysis were performed using the R program^56,57^. The major stages of the workflow were shown in Fig. 9.

### Raw data normalization and quality control

Each of the selected datasets was individually preprocessed using normalization approaches including the background correction to define and remove possible background noise, non-specific binding, and a log2 transformation. Raw intensity signals from Affymetrix (*.CEL files) were first normalized by applying either the GC Robust Multi-array Average (GCRMA) algorithm from the Bio-conductor R packages, gcrma, or Robust Multi-array Average (RMA) in oligo package^58–61^. Similarly, Agilent expression data was pre-processed by implementing background correction and quantile normalization (QN) from the R package LIMMA^62,63^. In both cases, highly variable genes at low intensities were removed to reduce false-positive rates. Furthermore, several plots and quality indicators were applied as a cyclic process prior to and after normalization to evaluate the quality of the datasets.

For quality assessment, open-source packages in Bioconductor such as affyPLM and simpleaffy for the Affymetrix platform, and LIMMA for Agilent platform were used^63–65^. Furthermore, relative log expression (RLE) plots, boxplots of deviations from gene medians, as the other quality assessment tool was applied to determine probe sets homogeneity. In addition, the correlation between arrays was evaluated using hierarchical clustering of arrays, principal component analysis (PCA) and heatmap.

### Intra-datasets filtering

After removing poor quality arrays, hgu133a.db and hgu133plus2.db R packages were used to annotate the probe IDs of the individual datasets to gene-level identifier (Entrez Gene IDs or official gene symbols) and to increase cross-platform concordance^66–69^. Genefilter package was used to filter out genes with different options of filtering including insufficient annotation, very low counts across all the arrays (low variance in intensities across samples) as well as control probe sets and other internal controls^70^. Finally, the normalized expression gene list of each dataset was integrated for downstream analysis.

### Integrative meta-analysis

Before merging datasets values and statistical analysis, the probe values of the same genes were averaged (summarized) to produce an expression value for each gene. Then datasets were reduced by Entrez Gene ID to cross-map genes among different platforms and extract the common genes from all studies. Integration of data from all platforms was done by using several cross-platform batch effect correction methods. We performed several exploratory analyses on the integrated data such as relative log expression (RLE) plots and PCA plots to assess the amount of batch (or unwanted variation) on the data.

Chemo-resistance and -sensitive AML groups were compared to identify differentially expressed genes regardless of sex, the French-American-British (FAB) classification and drug dosage. Besides, to uncover a more accurate set of differentially expressed genes involved in AML chemo-resistance two additional meta-analyses was performed using arrays of patient’s sample. Analysis on AML children was not performed due to lack of availability of pediatric patient’s arrays with complete remission. Finally, common DEGs among all meta-analyses were used.

### Statistical analysis

All statistical analysis was performed using R statistical software^57^. The CEL files were normalized and summarized with RMA method. Differential gene expression analyses for genes in sensitive and resistance cells were performed using linear regression models in the LIMMA R package^63^. *P *value < 0.05 and |fold change (FC)|> 1.5 were considered as the threshold of significance for DEGs. Benjamini–Hochberg (B–H) method was also used to analyze the results of t-statistics test and reduce false positive results.

### Predictive value analysis of hub genes

We constructed receiver operating characteristic (ROC) curve using GraphPad.Prism.9 software (https://www.graphpad.com) to evaluate the predictive accuracy of DEGs for chemo-resistance development. Predictive ability of the gene signature for clinical outcomes was evaluated by calculating the area under a ROC curve.

### Gene set enrichment analysis

Gene Ontology (GO) and pathway enrichment analysis were conducted for DEGs using the web-based enrichment analysis tool, Enrichr (https://maayanlab.cloud/Enrichr/), which contains > 180,000 curated gene sets in multiple categories from > 100 gene set libraries. DAVID (The Database for Annotation, Visualization and Integrated Discovery, https://david.ncifcrf.gov) was also used as another enrichment analysis tool which have two shared collections of libraries including the Gene Ontology (GO) (gene set database) and KEGG (pathway database, https://www.kegg.jp/kegg/kegg1.html) with Enrichr^71,72^.

### PPI network construction and module analysis

To represent the molecular interactions between various cellular processes through AML chemo-resistance, and also visualize the network of DEGs encoded proteins and protein–protein interactions (PPIs), we applied StringApp which is a visualization plug-in implemented in Cytoscape v3.7.0 environment^73^.

## Supplementary information

Supplementary Tables.
