# Supplementary material for: Meta-analysis of gene signatures and key pathways indicates suppression of JNK pathway as a regulator of chemo-resistance in AML
Source: Sci Rep. 2021 Jun 14;11:12485. doi: 10.1038/s41598-021-91864-2 (PMC8203646; doi:10.1038/s41598-021-91864-2)
Supplement: Supplementary file 1 — Supplementary Tables. [file 41598_2021_91864_MOESM1_ESM.pdf]

Meta-Analysis of Gene Signatures and Key Pathways Indicates Suppression of JNK Pathway as a Regulator of Chemo-Resistance in AML

Parastoo Modarres[1], Farzaneh Mohamadi Farsani[1+], AmirAbas Nekouie[2] and Sadeq Vallian[1*]

Supplementary Table S1. Enrichment analysis using the Enrichr platform for 34 DEGs associated with AML chemo-resistance. Ttwo well-known biological standard databases were used including: KEGG and Reactome.

Term	Category	P-value	Combined Score	Genes
Neurotrophin signaling pathway	KEGG_2019_Human	1.66E-06	399.5665812	JUN;SORT1;RPS6KA2;AKT3;MATK
Renal cell carcinoma	KEGG_2019_Human	2.18E-04	246.032917	JUN;EPAS1;AKT3
cAMP signaling pathway	KEGG_2019_Human	4.44E-04	97.78694163	JUN;GABBR1;AKT3;ARAP3
Toll-like receptor signaling pathway	KEGG_2019_Human	7.27E-04	137.5514418	JUN;AKT3;SPP1
Estrogen signaling pathway	KEGG_2019_Human	0.001610738	92.10941641	JUN;GABBR1;AKT3
Focal adhesion	KEGG_2019_Human	0.004627025	52.4755005	JUN;AKT3;SPP1
Fc epsilon RI signaling pathway	KEGG_2019_Human	0.005956694	96.5458987	ALOX5;AKT3
B cell receptor signaling pathway	KEGG_2019_Human	0.006477339	90.82413758	JUN;AKT3
ErbB signaling pathway	KEGG_2019_Human	0.009168483	70.24902579	JUN;AKT3
Colorectal cancer	KEGG_2019_Human	0.009376849	69.07680593	JUN;AKT3
Toll Like Receptor 10 (TLR10) Cascade Homo sapiens R-HSA-168142	Reactome_2016	1.11E-05	387.9069814	JUN;RPS6KA2;PELI2;S100B
Toll Like Receptor 5 (TLR5) Cascade Homo sapiens R-HSA-168176	Reactome_2016	1.11E-05	387.9069814	JUN;RPS6KA2;PELI2;S100B
MyD88 cascade initiated on plasma membrane Homo sapiens R-HSA-975871	Reactome_2016	1.11E-05	387.9069814	JUN;RPS6KA2;PELI2;S100B
TRAF6 mediated induction of NFkB and MAP kinases upon TLR7/8 or 9 activation	Reactome_2016	1.16E-05	381.3599264	JUN;RPS6KA2;PELI2;S100B
MyD88 dependent cascade initiated on endosome Homo sapiens R-HSA-975155	Reactome_2016	1.28E-05	368.8099564	JUN;RPS6KA2;PELI2;S100B
Toll Like Receptor 7/8 (TLR7/8) Cascade Homo sapiens R-HSA-168181	Reactome_2016	1.28E-05	368.8099564	JUN;RPS6KA2;PELI2;S100B
Toll Like Receptor 9 (TLR9) Cascade Homo sapiens R-HSA-168138	Reactome_2016	1.47E-05	351.2415045	JUN;RPS6KA2;PELI2;S100B
MyD88:Mal cascade initiated on plasma membrane Homo sapiens R-HSA-166058	Reactome_2016	1.75E-05	329.9061495	JUN;RPS6KA2;PELI2;S100B
Toll Like Receptor TLR1:TLR2 Cascade Homo sapiens R-HSA-168179	Reactome_2016	1.75E-05	329.9061495	JUN;RPS6KA2;PELI2;S100B
Toll Like Receptor TLR6:TLR2 Cascade Homo sapiens R-HSA-168188	Reactome_2016	1.75E-05	329.9061495	JUN;RPS6KA2;PELI2;S100B
Toll Like Receptor 2 (TLR2) Cascade Homo sapiens R-HSA-181438	Reactome_2016	1.75E-05	329.9061495	JUN;RPS6KA2;PELI2;S100B
Activated TLR4 signalling Homo sapiens R-HSA-166054	Reactome_2016	3.80E-05	249.5475669	JUN;RPS6KA2;PELI2;S100B
Toll Like Receptor 4 (TLR4) Cascade Homo sapiens R-HSA-166016	Reactome_2016	5.30E-05	220.7808776	JUN;RPS6KA2;PELI2;S100B
Toll-Like Receptors Cascades Homo sapiens R-HSA-168898	Reactome_2016	9.06E-05	180.9785517	JUN;RPS6KA2;PELI2;S100B
TRAF6 Mediated Induction of proinflammatory cytokines Homo sapiens R-HSA-168180	Reactome_2016	2.47E-04	231.7826783	JUN;RPS6KA2;S100B
MyD88-independent TLR3/TLR4 cascade Homo sapiens R-HSA-166166	Reactome_2016	5.93E-04	152.0007159	JUN;RPS6KA2;S100B
Toll Like Receptor 3 (TLR3) Cascade Homo sapiens R-HSA-168164	Reactome_2016	5.93E-04	152.0007159	JUN;RPS6KA2;S100B
TRIF-mediated TLR3/TLR4 signaling Homo sapiens R-HSA-937061	Reactome_2016	5.93E-04	152.0007159	JUN;RPS6KA2;S100B
MAPK targets/ Nuclear events mediated by MAP kinases Homo sapiens R-HSA-450282	Reactome_2016	0.001184328	299.8968905	JUN;RPS6KA2
Signaling by ERBB2 Homo sapiens R-HSA-1227986	Reactome_2016	0.002652714	171.7829446	AKT3;MATK
MAP kinase activation in TLR cascade Homo sapiens R-HSA-450294	Reactome_2016	0.00466778	115.1375666	JUN;RPS6KA2

Supplementary Table S2. Gene ontology terms for DEGs related to AML chemo-resistance provided by Enrichr based on P-value ranking for each category.

Term	GO Category	P-value	Combined Score	Genes
negative regulation of Rho protein signal transduction (GO:0035024)	GO_Biological_Process	2.52E-04	861.1431352	RIPOR2;ARAP3
cellular protein modification process (GO:0006464)	GO_Biological_Process	0.001262995	33.02666478	EPAS1;RPS6KA2;MGAT4A;AKT3;SPP1;MATK;PADI4
negative regulation of Ras protein signal transduction (GO:0046580)	GO_Biological_Process	0.001799795	224.9385169	RIPOR2;ARAP3
regulation of Rho protein signal transduction (GO:0035023)	GO_Biological_Process	0.004222337	123.7053823	RIPOR2;ARAP3
cholesterol homeostasis (GO:0042632)	GO_Biological_Process	0.004368495	120.7340787	ACSM3;INSIG1
sterol homeostasis (GO:0055092)	GO_Biological_Process	0.004368495	120.7340787	ACSM3;INSIG1
lipoxin biosynthetic process (GO:2001301)	GO_Biological_Process	0.010157909	555.2185649	ALOX5
hindlimb morphogenesis (GO:0035137)	GO_Biological_Process	0.010157909	555.2185649	AFF3
response to chemokine (GO:1990868)	GO_Biological_Process	0.010157909	555.2185649	RIPOR2
positive regulation of cellular extravasation (GO:0002693)	GO_Biological_Process	0.010157909	555.2185649	RIPOR2
SREBP signaling pathway (GO:0032933)	GO_Biological_Process	0.010157909	555.2185649	INSIG1
cellular response to chemokine (GO:1990869)	GO_Biological_Process	0.010157909	555.2185649	RIPOR2
negative regulation of lymphocyte migration (GO:2000402)	GO_Biological_Process	0.010157909	555.2185649	RIPOR2
interleukin-18-mediated signaling pathway (GO:0035655)	GO_Biological_Process	0.011841135	447.2023641	ALOX5
cellular response to interleukin-18 (GO:0071351)	GO_Biological_Process	0.011841135	447.2023641	ALOX5
nerve growth factor binding (GO:0048406)	GO_Molecular_Function	0.010157909	555.2185649	SORT1
arginine binding (GO:0034618)	GO_Molecular_Function	0.010157909	555.2185649	PADI4
protein kinase activity (GO:0004672)	GO_Molecular_Function	0.010789604	23.08424445	RPS6KA2;PELI2;AKT3;MATK
cAMP response element binding (GO:0035497)	GO_Molecular_Function	0.011841135	447.2023641	JUN
fatty-acyl-CoA synthase activity (GO:0004321)	GO_Molecular_Function	0.011841135	447.2023641	ACSM3
ribosomal protein S6 kinase activity (GO:0004711)	GO_Molecular_Function	0.011841135	447.2023641	RPS6KA2
phosphatidylinositol phosphate 4-phosphatase activity(GO:0034596)	GO_Molecular_Function	0.011841135	447.2023641	INPP4B
tau protein binding (GO:0048156)	GO_Molecular_Function	0.013521585	371.8308142	S100B
acyl-CoA ligase activity (GO:0003996)	GO_Molecular_Function	0.013521585	371.8308142	ACSM3
RAGE receptor binding (GO:0050786)	GO_Molecular_Function	0.01519926	316.4936999	S100B
neurotrophin binding (GO:0043121)	GO_Molecular_Function	0.01519926	316.4936999	SORT1
inositol trisphosphate phosphatase activity (GO:0046030)	GO_Molecular_Function	0.016874167	274.2892351	INPP4B
hydrolase activity, acting on carbon-nitrogen (but not peptide) bonds, in linear amidines (GO:0016813)	GO_Molecular_Function	0.016874167	274.2892351	PADI4
G-protein coupled receptor dimeric complex (GO:0038037)	GO_Cellular_Component	0.013521585	371.8308142	GABBR1

Supplementary Table S3. The expression of two multidrug efflux transporters genes, MDR1 (or ABCB1) and MRP1 (or ABCC1), in AML samples involved in the study. Data shows no differential expression between chemo-sensitive and chemo-resistance groups.

Accession	Group	ABCB1 Expression	ABCC1 Expression	treatment	platform	Gender	Age
GSM1278195	Resistance	6.392525293	8.55663226	Ara_DNR	Agilent	M	43
GSM1278196	Resistance	4.197349392	8.031078668	Ara_DNR	Agilent	F	61
GSM1278197	Resistance	6.705511723	8.167060668	Ara_DNR	Agilent	F	32
GSM1278198	Resistance	6.463722234	8.978745072	Ara_DNR	Agilent	F	43
GSM1278200	Sensitive	8.473923946	9.186866401	Ara_DNR	Agilent	F	43
GSM1278201	Sensitive	9.688854256	8.688948244	Ara_DNR	Agilent	M	50
GSM1278203	Sensitive	6.605658838	8.902160339	Ara_DNR	Agilent	F	33
GSM1278204	Sensitive	7.380205079	9.333275616	Ara_DNR	Agilent	F	44
GSM1278205	Sensitive	5.046742954	9.181699998	Ara_DNR	Agilent	M	50
GSM1278206	Sensitive	6.519826566	8.837074728	Ara_DNR	Agilent	F	18
GSM1278207	Sensitive	5.818359015	7.203262547	Ara_DNR	Agilent	F	44
GSM1278208	Sensitive	5.140046654	8.178725117	Ara_DNR	Agilent	M	44
GSM1277549	Resistance	2.978070014	7.945496536	AraC+Anthra	HG-U133_Plus_2	M	Chilhood
GSM1277551	Resistance	2.290297755	7.706025592	AraC+Anthra	HG-U133_Plus_2	M	Chilhood
GSM1277553	Resistance	2.524131953	7.193364563	AraC+Anthra	HG-U133_Plus_2	M	Chilhood
GSM1277554	Resistance	2.286647691	8.195704772	AraC+Anthra	HG-U133_Plus_2	M	Chilhood
GSM1277556	Resistance	2.286647691	6.349257618	AraC+Anthra	HG-U133_Plus_2	F	Chilhood
GSM1277557	Resistance	3.218586354	7.255220073	AraC+Anthra	HG-U133_Plus_2	M	Chilhood
GSM1277558	Resistance	2.379352435	7.59033488	AraC+Anthra	HG-U133_Plus_2	M	Chilhood
GSM1277559	Resistance	2.525807484	6.777112495	AraC+Anthra	HG-U133_Plus_2	M	Chilhood
GSM1277560	Resistance	2.286647691	8.020464716	AraC+Anthra	HG-U133_Plus_2	M	Chilhood
GSM1277561	Resistance	2.34926554	8.000281471	AraC+Anthra	HG-U133_Plus_2	M	Chilhood
GSM1277562	Resistance	2.30065544	7.392922065	AraC+Anthra	HG-U133_Plus_2	M	Chilhood
GSM1277563	Resistance	2.286647691	7.491117597	AraC+Anthra	HG-U133_Plus_2	F	Chilhood
GSM1277565	Resistance	2.286647691	7.604748063	AraC+Anthra	HG-U133_Plus_2	F	Chilhood
GSM1277567	Resistance	2.286647691	5.886008386	AraC+Anthra	HG-U133_Plus_2	M	Chilhood
GSM1277568	Resistance	2.429913165	6.745038687	AraC+Anthra	HG-U133_Plus_2	M	Chilhood
GSM1277569	Resistance	2.29876022	7.785129509	AraC+Anthra	HG-U133_Plus_2	F	Chilhood
GSM1277570	Resistance	2.286647691	7.106826653	AraC+Anthra	HG-U133_Plus_2	M	Chilhood
GSM2867943	Sensitive	2.482654434	8.769996342	Anthra+nucleoside Analog	HG-U133_Plus_2	F	63
GSM2867944	Sensitive	2.258032031	7.472987866	Anthra+nucleoside Analog	HG-U133_Plus_2	F	54
GSM2867946	Resistance	5.086906377	8.77331928	Anthra+nucleoside Analog	HG-U133_Plus_2	M	69
GSM2867949	Resistance	7.451693149	10.66320951	Anthra+nucleoside Analog	HG-U133_Plus_2	M	76
GSM2867952	Sensitive	2.258032031	8.308756374	Anthra+nucleoside Analog	HG-U133_Plus_2	M	30
GSM2867954	Resistance	2.258032031	9.295888704	Anthra+nucleoside Analog	HG-U133_Plus_2	F	49
GSM2867955	Sensitive	2.258032031	9.378052179	Anthra+nucleoside Analog	HG-U133_Plus_2	M	53
GSM2867959	Sensitive	3.544209166	7.884857654	Anthra+nucleoside Analog	HG-U133_Plus_2	F	56
GSM2867965	Sensitive	3.595764235	8.59987721	Anthra+nucleoside Analog	HG-U133_Plus_2	M	32
GSM1099774	Resistance	6.180020405	11.05757819	Ara_C+DNR	HG-U133A_2	NA	Childhood
GSM1099775	Resistance	4.413052553	10.72032548	Ara_C+DNR	HG-U133A_2	NA	Childhood
GSM1099777	Resistance	2.257781645	9.650320742	Ara_C+DNR	HG-U133A_2	NA	Childhood
GSM1099778	Resistance	2.257781645	9.262854546	Ara_C+DNR	HG-U133A_2	NA	Childhood
GSM1099780	Resistance	2.257781645	9.898768185	Ara_C+DNR	HG-U133A_2	NA	Childhood
GSM1099781	Resistance	2.257781645	9.405113861	Ara_C+DNR	HG-U133A_2	NA	Childhood
GSM1099782	Resistance	2.25714676	8.719512319	Ara_C+DNR	HG-U133A_2	NA	Childhood
GSM1099783	Resistance	2.355352439	8.615040091	Ara_C+DNR	HG-U133A_2	NA	Childhood
GSM1099784	Resistance	2.257781645	8.719592537	Ara_C+DNR	HG-U133A_2	NA	Childhood
GSM1099785	Resistance	2.257781645	8.204540161	Ara_C+DNR	HG-U133A_2	NA	Childhood
GSM1099789	Resistance	3.072815923	8.973645224	Ara_C+DNR	HG-U133A_2	NA	Childhood
GSM3265141	Resistance	4.924144103	10.32372076	Ara_C	HuGene-2_0-st	NA	NA
GSM3265143	Resistance	6.508744184	9.830004329	Ara_C	HuGene-2_0-st	NA	NA
GSM3265145	Resistance	5.58687792	10.12048055	Ara_C	HuGene-2_0-st	NA	NA
GSM3265147	Resistance	5.030558839	9.430270919	Ara_C	HuGene-2_0-st	NA	NA

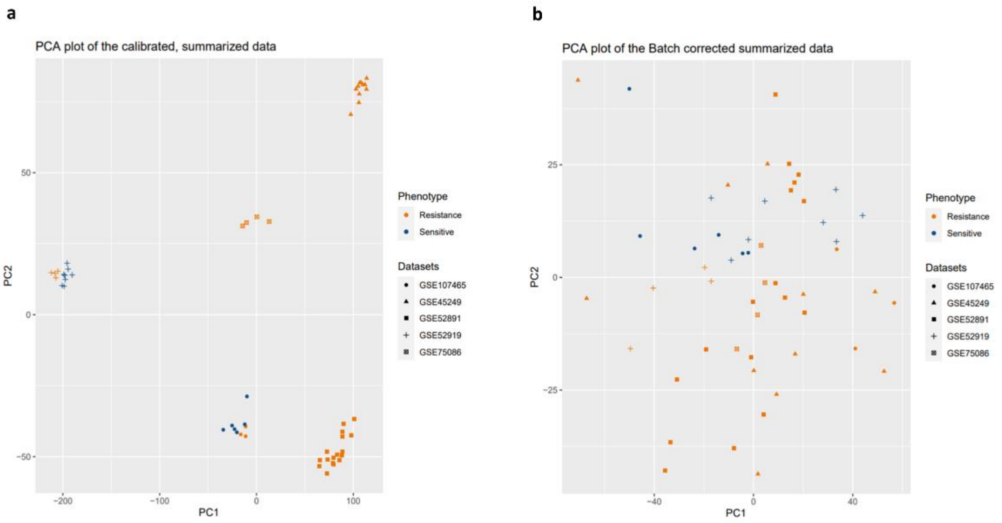

Supplementary Figure S1. Principal component analysis (PCA). PCA of the summarized data before and after batch correction was shown. The PCA plots illustrate the similarity of gene expression profiles among samples. Image generated using the ggplot2 package version 3.3.3 in R (https://www.R-project.org/).

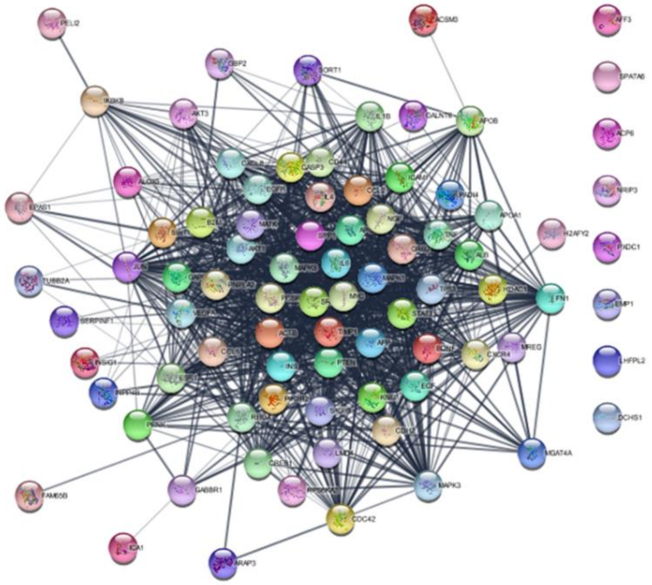

Supplementary Figure S2. Protein-protein interaction (PPI) network of 34 identified dysregulated genes associated with AML chemo-resistance. Functional enrichment analysis of DEGs were provided through stringApp tools in Cytoscape v3.7.0 (https://cytoscape.org/).
